# A burrowing frog from the late Paleocene of Mongolia uncovers a deep history of spadefoot toads (Pelobatoidea) in East Asia

**DOI:** 10.1038/srep19209

**Published:** 2016-01-11

**Authors:** Jianye Chen, Gaberiel S. Bever, Hong-Yu Yi, Mark A. Norell

**Affiliations:** 1 Department of Earth and Environmental Sciences, Columbia University, New York 10025; 2 Division of Paleontology, American Museum of Natural History, New York 10024; 3 Department of Anatomy, New York Institute of Technology, College of Osteopathic Medicine, New York 11568; 4 School of Geosciences, University of Edinburgh, Edinburgh EH9 3JW.

## Abstract

Fossils are indispensible in understanding the evolutionary origins of the modern fauna. Crown-group spadefoot toads (Anura: Pelobatoidea) are the best-known fossorial frog clade to inhabit arid environments, with species utilizing a characteristic bony spade on their foot for burrowing. Endemic to the Northern Hemisphere, they are distributed across the Holarctic except East Asia. Here we report a rare fossil of a crown-group spadefoot toad from the late Paleocene of Mongolia. The phylogenetic analysis using both morphological and molecular information recovered this Asian fossil inside the modern North American pelobatoid clade Scaphiopodidae. The presence of a spade and the phylogenetic position of the new fossil frog strongly support its burrowing behavior. The late Paleocene age and other information suggestive of a mild climate cast doubt on the conventional assertion that burrowing evolved as an adaptation to aridity in spadefoot toads. Temporally and geographically, the new fossil provides the earliest record of Scaphiopodidae worldwide, and the only member of the group in Asia. Quantitative biogeographic analysis suggests that Scaphiopodidae, despite originating in North America, dispersed into East Asia via Beringia in the Early Cenozoic. The absence of spadefoot toads in East Asia today is a result of extinction.

Frogs, the largest modern amphibian clade with more than 6500 species^1^, are both ecologically diverse and geographically widespread. Fossil frogs represent a unique window to trace their history in deep time, providing temporal, geographic, and occasionally, ecological information that is not evident in living species. Although the information fossils provide may be fragmentary and biased when studied alone, it becomes much more robust when combined with information from modern species and phylogeny.

One example is a group of frogs called Pelobatoidea. Commonly known as spadefoot toads, they are one of the best-known examples of fossorial frogs, inhabiting the most arid environments where amphibians survive^2^. Spadefoot toads gained their name because three pelobatoid clades (*Pelobates*, *Spea* and *Scaphiopus*) bear a distinct bony spade on their foot used in hindlimb burrowing. Other extant pelobatoids (Pelodytidae and Megophryidae) and some extinct species (e.g. *Eopelobates*), however, do not possess this spade. The homology of the spade has been questioned by molecular studies where *Pelobates* is more closely related to spade-less taxa than to *Spea* or *Scaphiopus*^3^,^4^. The burrowing behavior associated with the bony spade was assumed to be related to living in arid environments^5^,^6^. Geographically, modern spadefoot toads are distributed all across the Holarctic except East Asia^2^. A few fossil pelobatoids have been discovered from East Asia^7^,^8^,^9^, but the role of East Asia in the evolution of Pelobatoidea is still poorly understood due to the lack of incorporation of these fossils into a phylogenetic context.

Here we describe a spade-bearing frog from the late Paleocene of Tsagaan Khushuu, Mongolia. Its spade and other morphology strongly support it as one of the rare examples of spadefoot toads in East Asia. The new fossil species provides a valuable opportunity to better understand the evolutionary history of the spade and its associated burrowing behavior within pelobatoids, as well as revealing a cryptic history of the clade within East Asia. Combining the new fossil with other extant and extinct spadefoot toads in a phylogeny using morphological and molecular data, we aim to resolve the phylogenetic position of the new fossil, and to address 1) the homologous or homoplastic nature of the spade; 2) the relationships of the spade and its associated burrowing behavior with arid environments; and 3) the role of East Asia in the evolution of spadefoot toads.

## Systematic Paleontology

Amphibia Linnaeus, 1758.

Anura Rafinesque, 1815.

Pelobatoidea Bolkay, 1919.

Scaphiopodidae Cope, 1865.

*Prospea holoserisca* gen. et sp. nov.

## Etymology

*Prospea* means “before the North American spadefoot toad *Spea*”, and *holoserisca* means “silk”, referring to its discovery on the ancient Silk Road.

## Holotype

**Figure 1:**
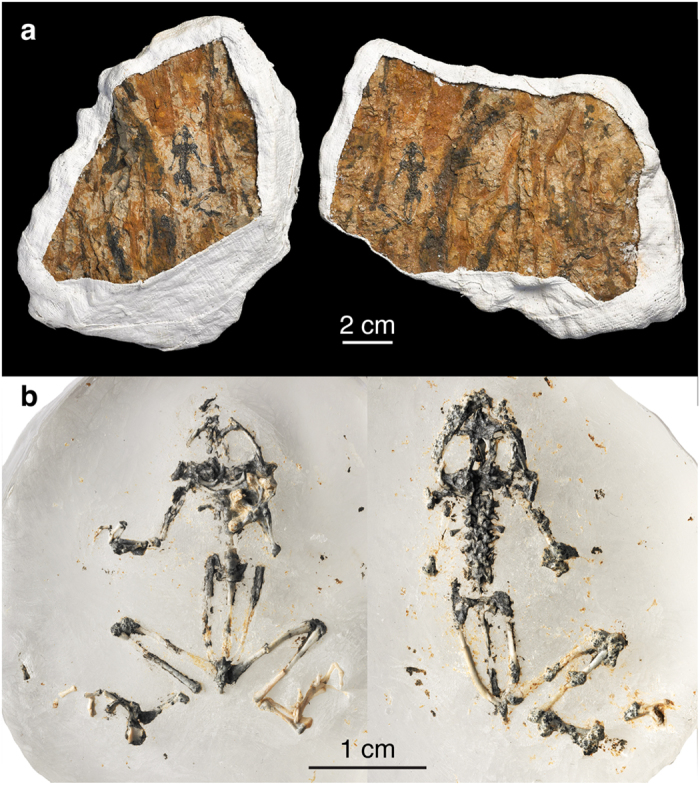
IGM 2/001, holotype of *Prospea holoserisca*. (**a**) The original specimen in rock matrix and jackets before preparation, preserved in part and counterpart; (**b**) the specimen in ventral and dorsal view after the preparation (Supplementary Experimental Procedures: fossil preparetion).

**Figure 2:**
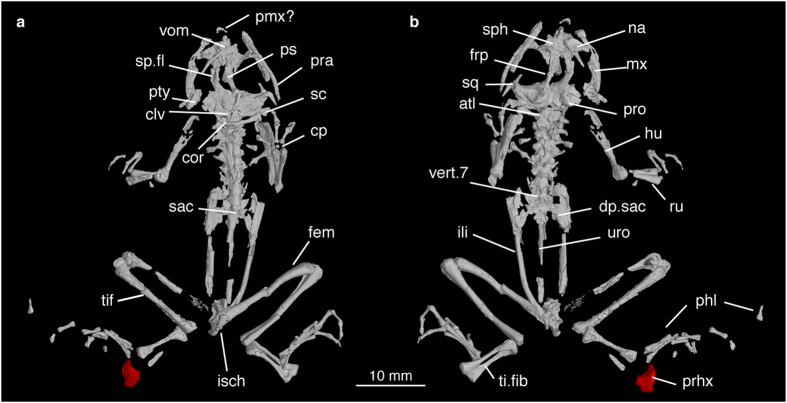
Digital reconstruction of the holotype of *Prospea holoserisca* (IGM 2/001) based on high-resolution CT scanning. The part and counterpart of the specimen was digitally joined together (also see Movie S1). Red color highlights the enlarged prehallux (bony spade). (**a**) Holotype in ventral view; (**b**) holotype in dorsal view. Anatomical abbreviations: atl, atlas; clv, clavicle; cp, carpal; cor, coracoid; dp.sac, diapophysis of sacral vertebra; fem, femur; frp, frontoparietal; hu, humerus; ili, ilium; isch, ischium; mx, maxilla; na, nasal; phl, phalange; pmx?, presumed premaxilla; pra, prearticular; prhx, prehallux; pro, prootic; ps, parasphenoid; pty, pterygoid; ru, radioulna; sac, sacral vertebra; sc, scapula; sp.fl, supraorbital flange of frontoparietal; sph, sphenethmoid; sq, squamosal; tif, tibiofibula; ti.fib, tibiale-fibulare; vert, vertebra; vom, vomer.

IGM 2/001 (Institute of Geology, Mongolia, Ulanbaatar, Mongolia), a nearly complete specimen preserved as part and counterpart in grey sandy clay (Fig. 1a). The rock matrix was later removed and the specimen was embedded in resin (Fig. 1b; see the Supplementary Experimental Procedures: fossil preparation). The two halves of the holotype were combined digitally to reconstruct the whole skeleton (Fig. 2; Supplementary Movie S1).

## Type locality and horizon

The new fossil was discovered at the “frog quarry”, Tsagaan Khushuu, Nemegt Basin of the southern Gobi Desert, Mongolia. It is the first frog fossil from this locality. The fossil is preserved in the grey lacustrine sandy clay of the upper phase of the Naran Member, Naranbulak Formation^10^. Biostratigraphic correlation based on mammalian fossils shows that the Naran Member is temporally equivalent to the Clarkforkian stage of North America (56.8 ~ 55.4 Ma). Conformably overlapping the Naran Member, the red beds of the Bumban Member are equivalent to the Wasatchian stage of North America^10^ (55.4 ~ 50.3 Ma). This framework places the frog fossil in the latest Paleocene, time equivalent to the Clarkforkian stage of North America at around 56 Ma.

## Diagnosis

The new fossil taxon is assigned to Scaphiopodidae based on the combination of the following characters: medial fontanelle between the frontoparietals present, supraorbital flange of the frontoparietal present, squamosal unsculptured, vertebrae procoelous, lateral margin of the scaral diapophysis convex, sacrum-urostylar articulation monocondylar, bony sternum absent, tibiale and fibulare fused proximo-distally, and metatarsal prehallux (bony spade) enlarged. It is unique within Scaphiopodidae in that the spade is triangular, instead of scaphoid as in *Scaphiopus*, or cuneiform as in *Spea*.

## Comparison

*Prospea holoserisca* differs from all extant pelobatoids (*Spea*, *Scaphiopus* and *Pelobates*, Megophryidae and Pelodytidae) in having an unfused sacrum and urostyle. Within Scaphiopodidae, it differs from *Spea* in having a zygomatic ramus of the squamosal, and from *Scaphiopus* in having a frontoparietal fontenelle and lacking sculpture on the skull roof. It differs from Pelobatidae in having unfused frontoparietals and hatchet-shaped diapophyses of the sacrum, and lacking an ossified sternum and the posteromedial element of the frontoparietal. It differs from Megophryidae and Pelodytidae in having an enlarged prehallux. Compared with other fossil spadefoot toads, *Prospea holoserisca* differs from *Macropelobates osborni* in its smaller size, a relatively longer urostyle than the presacral vertebrae, and lacking sculpture on the skull roof. It differs from *Eopelobates anthracinus* in having a dorsal acetabulum expansion of the ilium over the ischium and an enlarged prehallux, and lacking the posteromedial element of the frontoparietal and an ossified sternum. It differs from *Gobiates spinar* in having procoelous presacral vertebrae, and lacking sculpture on the skull roof, free ribs and transverse processes on urostyle. It differs from *Elkobatrachus brocki* in having more expanded sacral diapophyses and a larger prehallux, and lacking an ossified sternum and transverse processes on the urostyle.

## Description

*Prospea holoserisca* is a medium-sized frog, with a snout-pelvis length of 25 mm. It is much smaller than extant burrowing spadefoot toads (*Spea*, *Scaphiopus* and *Pelobates*). The ossification of carpal elements and humeral condyle (Fig. 2) indicates that it is a mature individual.

The skull is unsculptured and slightly longer than wide. The unfused nasals are crescent shaped, with an essentially straight anterolateral margin. The rostral process is developed as a distinct anterior projection towards the premaxilla. The frontoparietal has a lateral supraorbital flange, best seen from the ventral view, which is also present in extant pelobatoids (Fig. 2a). Similar to *Spea* but different from *Scaphiopus* and *Pelobates*, the paired frontoparietals are separated by a large fontanelle along the midline (Fig. 2b). There is no evidence of a posterior median element of the frontoparietal^11^,^12^. The squamosal is triradiate with three rami. The zygomatic ramus extends anteriorly towards, but does not contact, the maxilla (Fig. 2a). This is different from *Spea* where the zygomatic ramus is absent, and from *Scaphiopus* in which the zygomatic ramus contacts the maxilla. The otic ramus of squamosal is dorsoventrally flat and smooth, resembling *Spea*. In *Scaphiopus* and *Pelobates*, it is heavily ossified to form a bony plate with sculpture. The ventral ramus of squamosal extends posteroventrally to overlap the pterygoid. The sphenethmoid has an ossified septum nasi anteriorly and distinct lateral processes. The vomer is fragmentary, so is the parasphenoid, preserving only the anterior part of the cultriform process (Fig. 2a). The upper jaw consists of the premaxilla and maxilla, and the lower jaw consists of the dentary and prearticular (angulosplenial). Anteromedial to the maxilla, a small piece of bone probably represents the premaxilla (Fig. 2a). The tooth-bearing maxilla extends posteriorly to approximately two-thirds the length of the orbit without contacting the zygomatic process of the squamosal. In the lower jaw, the dentary is endentate. The prearticular is long, slender and slightly curved laterally.

The vertebral column consists of eight presacral vertebrae, the sacrum and the urostyle. As in extant spadefoot toads, all vertebrae have procoelous centra and imbricate neural arches. No ribs are observed, either absent or fused with presacral vertebrae. The atlas does not fuse with presacral II, and bears no transverse processes. Transverse processes of presacral II to IV are long and oriented laterally, those of presacral IV being the most robust. Transverse processes of presacral V to VIII are shorter and oriented anterolaterally. The sacrum bears a pair of widely dilated diapophyses with convex lateral margin, which is similar to *Spea* and *Scaphiopus* but different from *Pelobates*. The sacrum and the urostyle have a monocondylar articulation (Fig. 2a); in extant pelobatoids, the two bones are fused. The urostyle bears no transverse process or dorsal crest. It is slightly longer than the combined length of the presacrals.

The pectoral girdle preserves the clavicle, coracoid and the most medial part of the scapula. It is aciferal, as indicated by the non-parallel clavicle and coracoid (Fig. 2a). The clavicle is strongly bowed. Its distal end abuts the scapula instead of overlapping it. The coracoid is straight, with the distal end wider than the medial. The sternum is unossified, similar to *Spea* and *Scaphiopus*, but different from *Pelobates* with an ossified sternum. The humerus has a single enlarged distal condyle in articulation with the fused radioulna. Carpals are ossified (Fig. 2a), indicating its adult stage. The pelvic girdle consists of the ilium and ischium, with the pubis unossified. The ilium bears no dorsal ridge or dorsal tubercle, but has a prominent acetabular expansion that overlaps the ischium dorsally. The femur and tibiofibula are approximately equal in length. The tibiale and fibulare are fused at both ends, but separate along their shafts (Fig. 2). In the right foot, the prehallux is enlarged to form a triangular bony spade (Fig. 2). Among modern frogs, a single enlarged prehallux is characteristic of burrowing spadefoot toads (*Spea*, *Scaphiopus* and *Pelobates*).

## Results

### Phylogeny

**Figure 3:**
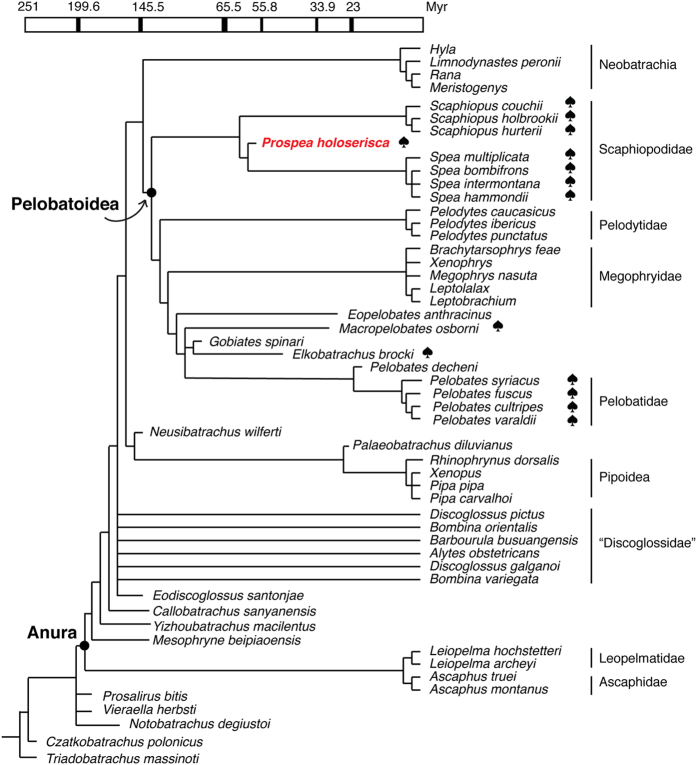
Evolutionary relationships (strict consensus) of modern and fossil “archaeobatrachian” frogs by combined parsimony analysis of 97 morphological characters and 9 genes, calibrated by fossil appearance (also see Supplementary Fig. S1 for the results from morphological data alone). Monophyly of each major modern clade is confirmed except for Discoglossidae. *Prospea holoserisca* is highlighted in red. The “spade” symbol marks the occurrences of bony spade within Pelobatoidea.

We sampled 97 morphological characters (five new characters) for 49 extant and extinct frogs, and nine genes (nuclear, mitochondrial and ribosomal) for 37 extant frogs. Analytical details are available in Supplementary Table S1 and Supplementary Experimental Procedures: phylogenetic analyses. Parsimony analysis of morphological and molecular data yielded 55 most parsimonious trees (MPTs), the strict consensus of which is shown in Fig. 3. We also performed a morphology-only analysis. In both analyses, the three burrowing clades in Pelobatoidea (*Pelobates*, *Spea* and *Scaphiopus*) do not form a monophyletic clade (Fig. 3; Supplementary Fig. S1). However, the North American *Spea* and *Scaphiopus* form a monophyletic Scaphiopodidae, supported by both analyses. This corroborates recent molecular phylogenies^3^,^4^, but contradicts some previous morphological phylogenies^13^,^14^,^15^.

Both analyses support *Prospea holoserisca* as a crown-group pelobatoid, in a sister-group relationship with North American *Spea* within Scaphiopodidae (Fig. 3; Supplementary Fig. S1). For other fossil pelobatoids, their phylogenetic positions are mostly unresolved in the morphological tree (Supplementary Fig. S1). Inclusion of the molecular data in the combined analysis greatly improves the resolution of the fossil taxa, along with the higher-level relationships within Pelobatoidea (Fig. 3). Two additional East Asian fossil species, *Gobiates spinari* and *Macropelobates osborni*, were both recovered as stem Pelobatidae. The Late Cretaceous *Gobiates spinari* from Mongolia was originally reported as a pelobatoid^8^, but was later excluded from the clade^16^,^17^. Our results support it as a crown-group pelobatoid. The middle Eocene *Elkobatrachus brocki* from North America was originally reported as the most basal pelobatoid^18^. Our new analyses, unexpectedly, recovered it as the sister taxon of *Gobiates spinari* (Fig. 3). For the abundant European fossil *Eopelobates*, we selected the better preserved late Oligocene *Eopelobates anthracinus* as a representative, and it occupies the most basal position along the pelobatid branch. The Oligocene *Pelobates dencheni* from Europe forms the sister-group of the four extant species of *Pelobates*.

Combining fossils, morphology and molecules helps to resolve a few controversial issues in the higher-level relationships of frogs, including the positions of some Mesozoic fossil taxa. Pelobatoidea and Neobatrachia form a monophyletic clade, which is different from morphological phylogenies^19^,^20^ but congruent with molecular phylogenies^4^,^21^. Monophyly of Discoglossidae^2^ is not recovered by our phylogeny. An unresolved polytomy exists between the presumed discoglossid fossil *Eodiscoglossus santonjae*^22^ and the four modern discoglossid genera (*Alytes*, *Discoglossus*, *Bombina* and *Barbourula*) (Fig. 3). Three frog species from the Early Cretaceous of China were coded individually: *Callobatrachus sanyanensis*, *Mesophryne beipiaoensis* and *Yizhoubatrachus macilentus*. Contrary to a recent study^23^, the three species do not form a monophyletic clade in our results, so we advocate their recognition as separate taxonomic units rather than lumping them collectively as *Liaobatrachus*.

### Biogeographic Analysis

**Figure 4:**
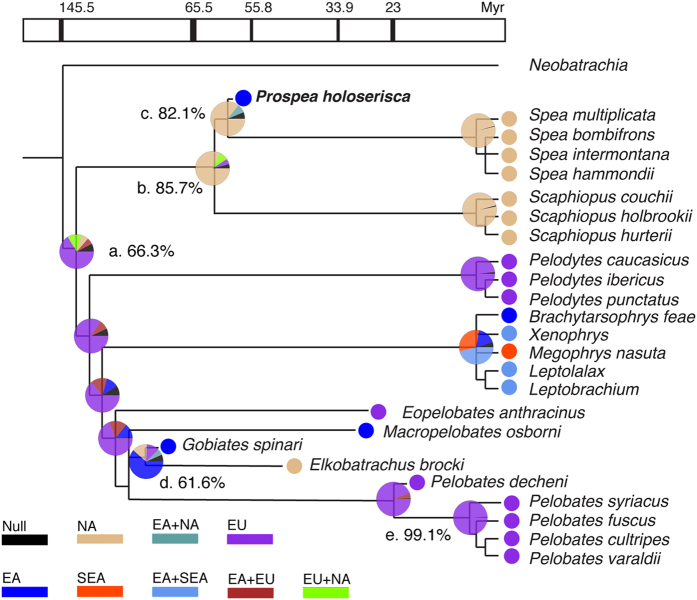
Historical biogeographic reconstruction of Pelobatoidea. The ancestral distribution is reconstructed for each of the internal node. The percentage following the node name represents the probability for the most likely distribution of the node. node (a) Pelobatoidea; (b) Scaphiopodidae; (c) stem *Spea*; (d) *Gobiates spinari* + *Elkobatrachus brocki*; (e) Pelobatidae. EA, East Asia; EU, Europe; NA, North America; SEA, Southeast Asia.

We performed quantitative historical biogeographic reconstructions to test the basis for the disjunctive distribution between fossils and their extant relatives. The 55 MPTs from the combined phylogeny were analyzed in RASP^24^ to identify the ancestral distribution for each internal node (Fig. 4). More analytical details are listed in Supplementary Table S1 and Supplementary Experimental Procedures: biogeographic analyses.

The base of Pelobatoidea (node a in Fig. 4) has the highest probability (p = 66.3%) for a European distribution. Crown-group Scaphiopodidae (node b) are native to North America (p = 85.7%). Because stem *Spea* (node c) is also North American (p = 82.1%), the appearance of *Prospea holoserisca* in Mongolia represents a dispersal event from North America to East Asia. The East Asian *Gobiates spinari* and North American *Elkobatrachus brocki* are sister groups, the ancestral node of which (node d) has the highest probability of East Asian origin (p = 61.6%). This indicates that *Gobiates spinari* was endemic to East Asia, and the occurrence of *Elkobatrachus brocki* in North America was due to dispersal from East Asia to North America. The node leading to *Macropelobates osborni* is unresolved, making both dispersal and vicariance possible. Crown-group Pelobatidae (node e) are endemic to Europe, so was the fossil *Pelobates dencheni*.

## Discussion

### Evolution of the spade and burrowing

With a late Paleocene age, *Prospea holoserisca* is the earliest fossil frog to express an enlarged bony spade (Fig. 2), and the earliest definite fossil frog adapted to hindlimb burrowing. In extant pelobatoids, hindlimb burrowers (*Spea*, *Scaphiopus*, and *Pelobates*) all bear the enlarged spade, whereas non-burrowing clades Megophryidae and Pelodytidae lack it. This led some morphological studies^2^,^13^,^14^,^18^ to assume monophyly of the three burrowing genera, with presence of spade as a synapomorphy. Molecular phylogenies^3^,^4^ later found that Pelobatidae (*Pelobates*) and Scaphiopodidae (*Spea* and *Scaphiopus*) are paraphyletic, but did not directly address how presence or absence of spade in fossils and their positions affect spade evolution. Our combined phylogeny confirmed the paraphyly of Pelobatidae and Scaphiopodidae, with fossil relatives representing both families. Plotting the presence of the spade on the tree (Fig. 3) suggests that the spade evolved convergently in Scaphiopodidae and Pelobatidae. The presence of the spade is not a synapomorphy of Pelobatoidea as a whole, despite the common name spadefoot toads. It is, however, still a shared derived character of all scaphiopodids, and independently, all extant pelobatids.

*Prospea holoserisca* is a member of the crown-group Scaphiopodidae. Anatomically, the enlarged spade suggests its ability to burrow. But more importantly, its burrowing behavior is supported by the extant phylogenetic bracket^25^ of *Spea* and *Scaphiopus* (Fig. 3). Modern scaphiopodids can withstand arid environments^2^ (e.g. the Sonoran Desert). Conventionally, adaptation to this environment has been considered to rely on burrowing and rapid larval development. A recent study on extant scaphiopodids has shown that rapid larval development does not relate to climate, but to genome size and phylogeny^26^. Our study further casts doubt on the relationships between burrowing and aridity^5^,^6^. The mild climate of the late Paleocene^27^ and the lacustrine deposits that preserve *Prospea holoserisca* suggest that it did not live in an arid environment. This indicates that burrowing is an exaptation^28^ instead of adaptation to arid environments in scaphiopodids. In other words, burrowing did not evolve because of the aridity, but subsequently it helps the frogs to survive when the environments became arid.

### Hidden History of spadefoot toads in East Asia

*Prospea holoserisca* is the earliest scaphiopodid worldwide, predating the early Eocene *Scaphiopus guthriei* from North America^29^. It provides a new time calibration for the origin of Scaphiopodidae at around 56 Ma. Extant scaphiopodids are endemic to North America, and very few fossils from other continents can be included in the group^30^. In Asia, *Prospea holoserisca* is the only fossil scaphiopodid as suggested by our phylogeny. *Macropelobates osborni* was proposed to belong to Scaphiopodidae^30^,^31^, but our phylogeny shows that it is more closely related to *Pelobates*.

The presence of *Prospea holoserisca* in Mongolia reveals a previously unknown dispersal event from North America to East Asia. The upper boundary of the dispersal is defined by the occurrence of the fossil at approximately 56 Ma. The lower boundary, we suggest, should be placed at the beginning of the Paleocene at 66 Ma^32^. This is because most land animals and plants share little similarities between North America and East Asia until the latest Cretaceous, which suggests that Beringia–the only land bridge connecting the two continents–may not be passable for the most time of the Creteceous^33^. Being the first epoch after the K-Pg extinction, the Paleocene is a key interval that shaped the biogeographic distribution of the modern fauna. The Paleocene/Eocene Thermal Maximum (PETM) is one of the best-known intervals for large-scale faunal exchange^34^,^35^. The discovery of *Prospea holoserisca* suggests that smaller scale faunal exchanges such as the dispersal of spadefoot toads started to occur before the climate became extremely warm during the PETM.

The role of East Asia in the evolution of Pelobatoidea has been long overlooked. Molecular analyses are restricted to modern taxa and thus cannot reconstruct the full biogeographic history of the clade due to their absence in East Asia today. Incorporating fossils with extant taxa and molecular data provides an important opportunity to address this problem. Our analyses demonstrate that both scaphiopodids (*Prospea holoserisca*) and stem pelobatids (*Gobiates spinari* and *Macropelobates osborni*) inhabited East Asia by the Early Cenozoic. The pattern of their subsequent extinctions deserves future study, but dramatic climate change in East Asia during the Cenozoic^27^ may have been an important factor.

## Additional Information

**How to cite this article**: Chen, J. *et al.* A burrowing frog from the late Paleocene of Mongolia uncovers a deep history of spadefoot toads (Pelobatoidea) in East Asia. *Sci. Rep.* **6**, 19209; doi: 10.1038/srep19209 (2016).

## Supplementary Material

Supplementary Information

Supplementary Table S1

Supplementary Movie S1
