# Supplementary material for: A burrowing frog from the late Paleocene of Mongolia uncovers a deep history of spadefoot toads (Pelobatoidea) in East Asia
Source: Sci Rep. 2016 Jan 11;6:19209. doi: 10.1038/srep19209 (PMC4707494; doi:10.1038/srep19209)
Supplement: Supplementary Information [file srep19209-s1.pdf]

Supplementary Information for:

A burrowing frog from the late Paleocene of Mongolia uncovers a deep history of spadefoot toads (Pelobatoidea) in East Asia

CHEN, JIANYE[a,b,*]
jchen@amnh.org

BEVER, G. S.[b,c]
gbever@nyit.edu

YI, HONG-YU[d]
v1hyi@staffmail.ed.ac.uk

NORELL, MARK A.[a,b]
norell@amnh.org

a. Department of Earth and Environmental Sciences, Columbia University, New York 10025;
b. Division of Paleontology, American Museum of Natural History, New York 10023
c. Department of Anatomy, New York Institute of Technology, College of Osteopathic Medicine, New York 11568
d. School of Geosciences, University of Edinburgh, Edinburgh EH9 3JW

* To whom correspondence should be addressed.

Supplementary Table S1. Data sampling for the phylogenetic and biogeographic analyses. (see a separate excel file for the table)

Supplementary Movie S1. Digital reconstruction of the holotype of *Prospea holoserisca*. (see a separate .mov file for the movie)

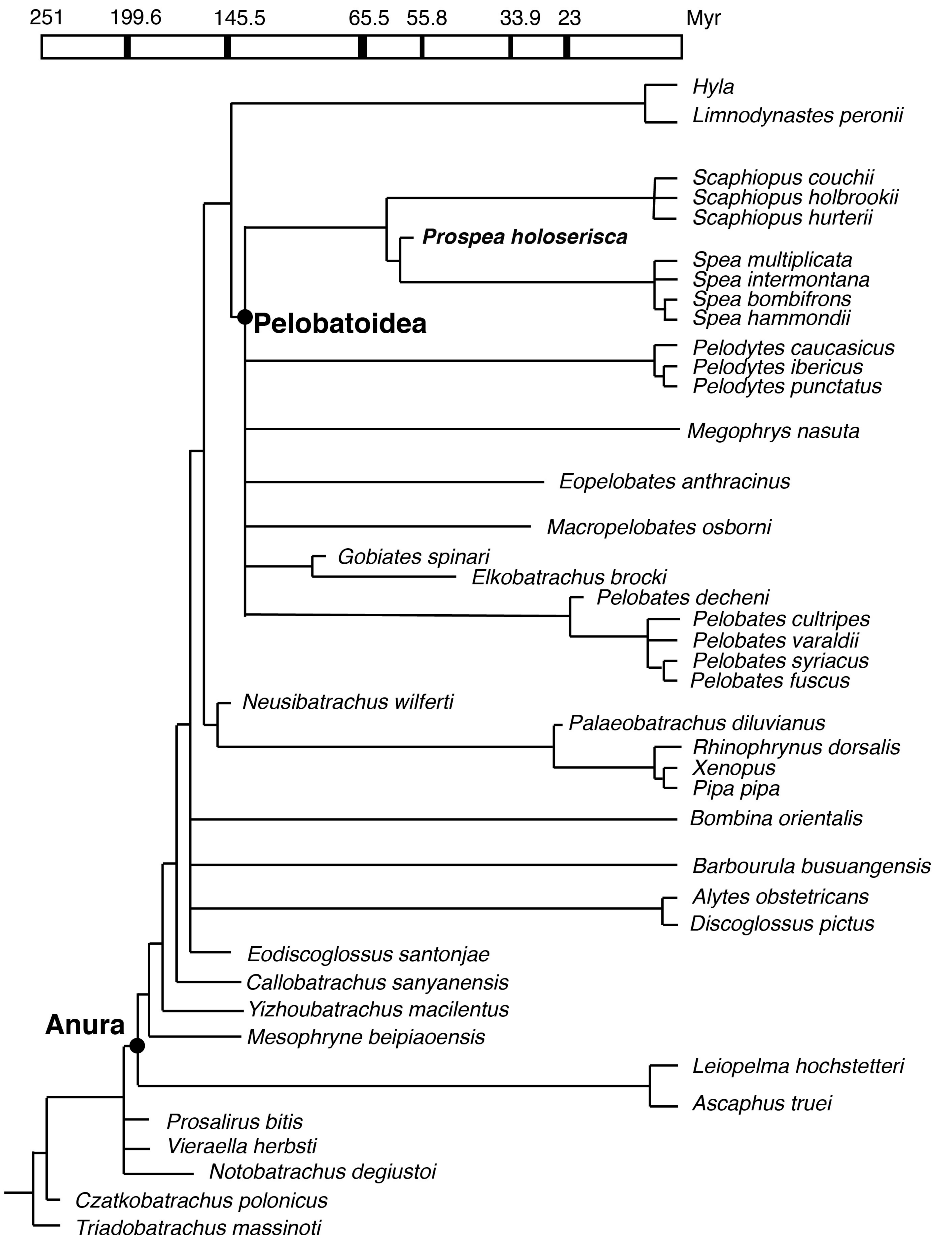

Supplementary Figure S1. Strict Consensus of 106 most parsimonious trees from the morphology-only analysis. The tree is calibrated by fossil appearances. The sister-group relationship of the new fossil *Prospea holoserisca* (in bold font) and *Spea* are supported, the same as the combined analysis (Fig. 3).

Supplementary Experimental Procedures

Fossil Preparation: The specimen was collected split in 2 blocks and consolidated in the field with Butvar B-76 terpolymer of vinyl butyral, vinyl alcohol and vinyl acetate. The field coating was removed by swelling with acetone and picking off with a needle. Both halves were partially prepared using needles and then embedded in Epo-Tek 301-2 epoxy (diglycidyl ether of bisphenol A resin and aliphatic amine hardener). After embedding, both halves were prepared with an airscribe, grinder and scraping with a needle. The specimen was consolidated in spots with Aron Alpha 201 ethyl-2-cyanoacrylate.

CT scanning and digital reconstruction: The specimen was scanned under 180kv nano tube using the GE phoenix v|tome|x s240 system CT machine in American Museum of Natural History. It was digitally reconstructed and rejoined together to form a single skeleton using VGStudio Max 2.2.

Phylogenetic Analyses. Data sampling includes 49 taxa coded for 97 morphological characters, and nine gene sequence data (5436 base pairs) (Supplementary Table S1). Triassic stem frog *Triadobatrachus massinoti* was chosen as outgroup. Some pelobatoid fossils were not included in this analysis, either due to their isolated nature (e.g. *Spea neuter*), or due to their unavailability for the authors at the time of this research (e.g. *Macropelobates linquensis*). The morphological characters used in the analysis were mostly modified from previous studies[13,14,18-20,s1], but also contain five new characters. All characters were unordered and weighted equally. Coding for morphological characters relied on museum specimens and publications (as detailed in Supplementary

Table S1). Gene sequences were downloaded from Genbank[s2] and aligned using MUSCLE[s3] under default settings. For *Leptolalax*, *Xenophrys*, *Xenopus*, *Hyla*, *Leptobrachium*, *Rana* and *Meristogenys*, because different species were sequenced across different genes, we merged the gene data of different species into the same genus in our analysis. The analysis was performed under Maximum Parsimony criterion using POY 4.1.2[s4]. Tree searching methods include tree building, swapping using TBR, perturbation using ratchet, and tree fusing. Besides the combined analysis as shown in the paper, we also ran a morphology-only analysis. It recovered 106 MPTs and a strict consensus (Supplementary Figure S1) that, while had less overall resolution than the combined analysis, did also recover a monophyletic Pelobatoidea and *Prospea* as the sister taxon to *Spea*. A few differences between the morphological tree and the combined tree include: 1) the morphological tree does not resolve the root node of Pelobatoidea, leaving a polytomy among Pelobatidae, Scaphiopodidae, Megophryidae and Pelodytidae; 2) it supports a monophyletic relationship of *Pelobates syriacus* and *Pelobates fuscus*, which is not supported by the combined analysis; 3) it supports a monophyletic relationship of *Alytes* and *Discoglossus*, which is not supported by the combined analysis.

Biogeographic Analyses. We performed the S-DIVA reconstruction and Bayesian MCMC analyses using RASP[24]. Two analyses bear similar results, so we only show the results from the Bayesian analysis. 55 MPTs were used to generate a “condensed tree” in RASP, which was subsequently used for both analyses. Four distribution units are defined and assigned to each taxon: East Asia (northern China, Mongolia and East Siberia), Europe, North America and Gondwana (which includes South East Asia). For

both analyses we used the default settings, and for the Bayesian analysis we set the root distribution to follow the outgroup.

Morphological Data Matrix

Triadobatrachus massinoti

000??0?1000000????0?0?0000?????0?00000000?0??0?0000?00000000???000?00??0??0

00001000?0?000????????

Czatkobatrachus polonicus

???0?0???10??????????1????????010??1

?00??????????????

Prosalirus bitis

???????1??00???11?????????1??????0?0????0??0???0?0???10{12}???0???1?0???1?????

01??1?20?1??0?????????

Vieraella herbsti

1?0?01?1011001???001??1001101101?010?????00??1?00?{12}?0?0?0?0?0????0?001?0

???01??1??0?1?0??????????

Notobatrachus degiustoi

000000?101{12}?11001000111001?001010000?1?00000?2?0021?0101000{12}0??1000

001?0??0010?1020?1??0?????????

Ascaphus truei

00111?010220?10?01?111100100?0?1011100001000?20012200102000000001011001?0

020111010?00111101?000000

Leiopelma hochstetteri

00111?010220?20?01?1111001001201011101001000?20012200102100000010000001?0

0301110102001111101?100000

Alytes obstetricans

00001?010210?21?10?1201001101201111100010000?30110200112?10?010101100001

031111011200120101?1000?0

Barbourula busuangensis

00000001001002100001201001101201111100100000?301002?0112?20?0001011?10?1

031111010100100101?1000?0

Bombina orientalis

00000101021012101001301001101201111100001000030100200112?2000001011100?1

031111010{01}00100101?1000?0

Discoglossus pictus

000000010{12}1012101001201101101201111100110000030100200112110?010101110

0010311110112101201?01?1000?0

Eodiscoglossus santonjae

000001?1001001??100?201001?0????????????0?00?3010?2?01{01}2110?0????1??00?1

????11?102?01?01?1???????

Callobatrachus sanyanensis

000?0??10?20?2??100?201001101201????????0000?2010?2?0112110?0??1011??0?0???

111?1?2??1?01?1??0????

Mesophryne beipiaoensis

000?0??1?????2?0000??01101?0?????11?0?0?000??2020?2?01120?0????10??0010????

110?1?2?01?01?0??0?0??

Pipa pipa

10011{01}{12}110210221011011300111???10211111201{01}1?31100321122132?101

1001{01}010102?111010021100111?201000

Xenopus

{01}02100011020123101101130011{12}???102111012010103{01}102321122132?101

10120100102?111010021110111?201000

Rhinophrynus dorsalis

100000?11021?21?00111020011011?10210020011110301004?1102?12??001101?01?20

01111011200100111?210010

Palaeobatrachus diluvianus

10000??110?0?21?00102{01}10011?1??10210?0?00001?312023?0112?12????1?11??1?

1??011{01}?1?2??1?0111???????

Neusibatrachus wilferti

0??????11??0????1?0??0?????01??1021??0?00?0??31202{34}20102112?0???0111000?

????1??1????1????????????

Hyla

{01}0011?010220?21?10?12010101011001111?1020?01?312024?0112112?{01}101100

1010001?11121010?100111?210011

Limnodynastes peronii

10010?0101?0?21?10?120100110110011111?1020101?312114{12}0102112?110110000

101{01}1?11121021?1001{01}1?210011

Yizhoubatrachus macilentus

00????0101?1???1010??0?????0????001?01?0000??2?1????0112?12?0???0111001????1

1??1????1??0????0????

Pelodytes caucasicus

001111010010 12?1000120100110120111010001000103020041112 2?2??01011001011?

1??111111001002?1?210100

Pelodytes punctatus

0010000102201 2?1000120100110120111110001000103020141112 2?2??01010101011?

1??11?1111001002?1?210100

Pelodytes ibericus

00111001022012?100012010011012011111000100010302014111 22?2??01010101011?

1??11?1111001002?1?210100

Megophrys nasuta

00010?0100?1?2??10?1201010101101111 10?020001130200410 1?2?{03}1?0201100?01

?01??11101?1?0100111?21000?

Elkobatrachus brocki

?0?10??10210?2?010??2?????10?????1100?????01130?0?411112120?0??1101001?011?

11??1???01?00?0??1????

Prospea holoserisca

10010??102111{12}???0012?100110???1?11?010???01?3020?{34}11122021?0??1??01

001?01111?01?20?1??1?22?1????

Spea multiplicata

0001002102111 2??1011212001101211111101020001?30200411112 03{12}0010110010

01101?1110112001001122?10100

Spea hammondii

00110?2102?112??10?121200110?211?1?1010?000113020041111203100101100100 1?0

1?11?01?200100??22?10100

Spea intermontana

000100210121 12??10112120011012110111010200011302004111120320010110010 01?

01?1110112001001122?10100

Spea bombifrons

001101210211 12?010112120011012111111010200011302004111120310010110010 01?

01?1110112001001122?10100

Scaphiopus hurterii

020100210021 02??1001211111101211111101020001130200411?120??0010110010 01?

01?11?01?200100??21?10100

Scaphiopus holbrookii

020100210011 02??10012111111012111111010200011302004111120320010110010 01?

01?11?011200100??21?10100

Scaphiopus couchii

020100210011 02??10112111111012111111010200011302004111120320010110010 01?

01?11?01?200100??21?10100

Macropelobates osborni

010????1001112??????20111110?????1??0?0?0?01?3020?4?1122022?0??11????1?????

11??112011?00?21?1????

Eopelobates anthracinus

00010?21001102???00?20111110?10?111101020?0?13?20?4?1122022????1?00??1??1?

?11??102??1?000????????

Pelobates decheni

010000?1001102???00?201110??????????????????130?0?41?122022?0??1???????0???1

1??1?2?01??0?????????

Pelobates varaldii

01000?2110?102??10?1201110101001111 10?02000113020041112203200101100101 1?

11?11?01?200100??21?10100

Pelobates cultripes

01000121100102?010012011101010011111010200011302004111220{23}20010110?10

11011?1110112{01}01001?21?10100

Pelobates fuscus

01000?211011021?100120111{01}101001111101?22001130200411122032001011 0010

11011?111011200100{01}121210100

Pelobates syriacus

01000?2110?102??10?120111010100111 1101?22001130200411122032001011001011?

11?11?011200100??21?10100

Gobiates spinari

02000021021011?0??0020111110?0?111?101???0??13000?2?1112110?0??111010?????
??1??1?0?01????0???????

Morphological Character List

1. Shape of the skull in dorsal aspect: skull apparently wider than long (0); or roughly as long as wide, or longer (1).

   Remarks: Modified from [19] (character 1) and [20] (character 1). The length of the skull is measured from the tip of snout to the foraman magnum, and the width is measured from its widest part, usually at the angle of jaws. In *Triadobatrachus* the skull is wider than long, and this is considered as the primitive condition.

2. Sculpture on dermal skull roof: absent or only weakly present (0); or present, with a pitted pattern (1); or present, with a grooved pattern (2).

   Remarks: Modified from [19] (character 3) and [20] (character 2). *Triadobatrachus* has low irregular rugosities on the anterior part of the frontoparietal, but not on other dermal roofing bones. Extensive sculpture of dermal skull roof can be seen in *Pelobates* as having a pitted pattern, and in *Scaphiopus* as having a grooved pattern.

3. Medial contact of nasals: contact present (0); or contact absent (1); or nasal fused medially (2).

   Remarks: Modified from [20] (character 3). *Triadobatrachus* is reconstructed to have two nasals with a medial articulation, and this is considered to be primitive.

Condition 1 is seen in living leiopelmatids and pelodytids, and condition 2 is seen in *Xenopus*.

4. Anterolateral margin of nasal: nasal with a concave anterolateral margin for embracing the narial opening (0); or nasal more circular, with essentially a straight anterolateral margin, not embracing the narial opening (1).

   Remarks: Modified from [19] (character 5). The polarity is tentative, because *Triadobatrachus* has no anterior part of nasal preserved. In Jurassic frog *Vieraella* and *Notobatrachus*, the nasal has a concave anterolateral margin, so this is considered primitive. Condition 1 is seen in most pelobatoids.

5. Distinct rostral process of nasal: present (0); or absent (1).

   Remarks: Modified from [20] (character 5). When present, it is a moderately developed process extending anteriorly towards the premaxilla along the midline[19] and above the septum nasi. Polarity of this character is tentative, because *Triadobatrachus* has no anterior part of nasal preserved. Distinct rostral process of nasal is seen in *Vieraella* and *Notobatrachus*, so it is considered as the primitive condition.

6. Extent of posterior divergence of nasals: divergence minimal, involving less than half the length of nasals (0); or divergence extensive, involving over half the length of nasals (1).

   Remarks: Modified from [13] (character 9). The nasals start to diverge posteriorly to variable degree. It can either involve only the posterior most part (condition 0), or involve as much as about the whole length of the nasal. The

posterior edge of nasal in *Triadobatrachus* stays close to the midline[s5], so the minimal divergence is considered as the primitive condition.

7. Ossification of septum nasi: septum nasi cartilaginous (0); or septum nasi bony posteriorly, extending about one-half the length of the nasals (1); or septum nasi bony along most of the length of the nasals (2).

   Remarks: Modified from [19] (character 15). Polarity is tentative. The septum nasi is the midsagittal wall on the sphenethmoid that separates the nasal organs from each other. Coding for living taxa follows [19]. Coding for *Gobiates* follows [17].

8. Fusion of frontal and parietal: frontal and parietal remain separate (0); or fused to form frontoparietal (1).

   Remarks: Modified from [20] (character 6). The derived condition is a salientian synapomorphy[s6].

9. Fusion of two frontoparietal medially in adults: frontoparietal paired without fusion (0); or azygous frontoparieal present due to fusion (1).

   Remarks: Modified from [19] (character 21) and [20] (character 7). A Pair of frontoparietals is seen in *Triadobatrachus* and is considered as the primitive condition. The azygous frontoparietal is independently evolved within pipids and pelobatids. Different from living *Pelobates*, fossil taxa *Macropelobates* and *Eopelobates* have paired frontoparietals.

10. Dorsal exposure of frontoparietal fontanelle: fontanelle not exposed (0); exposed 50% of its length or less (1); or exposed more than 50% of its length (2).

Remarks: Modified from [19] (character 22) and [20] (character 8). *Triadobatrachus* has no fontanelle exposed dorsally between the frontoparietals, and it is considered as the primitive condition.

11. Posterolateral process (margo prootica) of frontoparietal: well developed and wing-like (0); or poorly developed (1); or completely absent (2).

    Remarks: Modified from [20] (character 9). The wing-like posterolateral process occurs in *Triadobatrachus* and *Prosalirus*. This is considered as the primitive state. Other frogs either have a small process or completely lack the process.

12. Supraorbital flange of frontoparietal: absent (0); or present (1).

    Remarks: Modified from [19] (character 25) and [20] (character 10). When present, the supraorbital flange is the lateral expansion of the frontoparietal to roof over the orbit. *Triadobatrachus* lacks such a flange, so the absence is considered as primitive.

13. Contact between frontoparietal and nasal: contact present (0); contact absent (1).

    Remarks: Modified from [S7] (character 4). *Triadobatrachus* has the nasal contacting the frontoparietal, so this is considered as the primitive condition.

14. Formation of prootic-occipital region: by prootic-exoccipital-opisthotic complex (0); by prootic-exoccipital without fusion (1); or by fused prootic-exoccipital (2).

    Remarks: Modified from [20] (character 11). *Triadobatrachus* is reported to have the opisthotic in the ear region, a condition similar to salamanders[s5]. Presence of opisthotic is considered as the primitive condition. Other frogs only retain the prootic and exoccipital, either as discrete elements (as in

Notobatrachus, *Vieraella*, and *Ascaphus*) or fused together. Coding for *Gobiates* follows [17].

15. Perilymphatic foraman: double foramina open on medial capsular wall (0); or double foramina present on posterior wall of otic capsule (1); only superior perelymphatic foramen present (2); or only inferior foramen present (3).

    Remarks: Modified from [20] (characters 12). In urodeles, the perilymphatic foramina are absent on the posterior wall of the otic capsule (open on the medial wall, instead). A similar condition is seen in *Ascaphus* and *Leiopelma*[s7]. A single foramen in *Notobatrachus* is interpreted as the jugular foramen[s8].

16. Width of alary process of premaxilla: thin, with one fouth or less width of premaxilla (0); one third or greater width of premaxilla (1).

    Remarks: Modified from [13] (character 12). The polarity is tentative.

17. Palatine process of premaxilla: absent or barely present (0); or well developed (1).

    Remarks: Modified from [19] (character 52) and [20] (character 13). The polarity is tentative due to unknown condition in *Triadobatrachus*. When present, it is a posterior projection from the medial end of the pars palatina of the premaxilla. Because the premaxilla in salamanders lacks such a projection, the absence is considered as primitive.

18. Premaxilla-maxilla articulation: posterior process of premaxilla absent (0); or present (1).

    Remarks: Modified from [20] (character 14). The polarity is tentative due to unknown condition in *Triadobatrachus*. When present, it is an elongate and pointed posterior projection from the pars palatina of the premaxilla. In living

taxa, condition 1 is seen in *Ascaphus*, *Leiopelma*, and probably independently in *Pipa* and *Xenopus*.

19. Posterior extent of maxilla: maxilla long, extending posteriorly for most of the length of the orbit (0); or maxilla relatively short, not extending posteriorly beyond half the length of orbit (1).

    Remarks: Modified from [13] (character 18). *Triadobatrachus* has a long maxilla extending to the posterior extremity of the orbit, so the long maxilla is considered the primitive condition in frogs. In living taxa, a short maxilla is seen in *Pipa*, *Xenopus*, *Rhinophryus* and *Spea*.

20. Premaxilla-nasal articulation: articulation present (0); or articulation lost with separation of the two elements (1).

    Remarks: Modified from [20] (character 15). In salamanders, the alary process of the premaxilla contacts or overlaps the nasal, so the contact between the two bones is considered as primitive.

21. Prefrontal and anterior margin of the orbit: prefrontal present, maxilla and nasal excluded from the anterior margin of the orbit (0); prefrontal lost with maxilla and nasal forming the anterior margin of the orbit (1); nasal forming most of the anterior margin of the orbit (2); or anterior ramus of pterygoid excluding maxilla from the anterior margin of the orbit (3).

    Remarks: Modified from [20] (character 16). Prefrontal is present in salamanders and *Triadobatrachus*, so it is considered as the primitive condition in frogs. Other frogs either have the nasal alone or the nasal together with the maxilla to form the

anterior border of the orbit. *Bombina* is unique in having a long anterior ramus of pterygoid to exclude maxilla from the margin of orbit.

22. Quadratojugal: present (0); or absent (1).

    Remarks: Modified from [19] (character 62) and [20] (character 17). Polarity of this character is tentative, following [20] but in contrast with [19]. Condition in *Triadobatrachus* cannot be decided[s5].

23. Shape of squamosal: as a simple horizontal bar (0); or triradiate and T-shaped (1); or nontriradiate with loss of zygomatic ramus (2); or funnel shaped in fusion with tympanic annulus (3).

    Remarks: Modified from [19] (character 40, 41, 42) and [20] (character 18). *Triadobatrachus* and salamanders all possess a horizontal bar-shaped squamosal, and this is considered the primitive condition. Most frogs have a triradiate squamosal with an otic ramus, a zygomatic ramus and a ventral ramus. In *Spea* and *Rhinophryus* the zygomatic process is reduced. In *Pipa* and *Xenopus* the squamosal is elaborated into a funnel-shaped structure to house the columella[19].

24. Squamosal-maxilla contact: absent (0); or contact present (1).

    Remarks: Modified from [19] (character 43) and [20] (character 19). Most frogs have a short zygomatic ramus of squamosal that is free from bony contact. In living taxa, *Discoglossus*, *Pelobates* and *Scaphiopus* have the derived condition.

25. Expansion of otic ramus of squamosal in lateral view: not expanded (0); or otic ramus expanded and deep (1).

    Remarks: Modified from [20] (character 20). In some taxa, the otic ramus of the squamosal is expanded and elaborated to form a lateral wall.

26. Medial articulation of squamosal: squamosal medially in contact with dermal skull table (0); or squamosal not in contact with dermal skull table, but articulating with the crista prootica (1).

    Remarks: Modified from [20] (character 21). Although in the holotype of *Triadobatrachus*, the squamosal and frontoparietal are disarticulated from each other, they might be in contact in its original form. The contact between squamosal and the skull table is considered as primitive.

27. Sphenethmoid: bilaterally paired (0); or single (1).

    Remarks: Modified from [19] (character 17) and [20] (character 22). The paired sphenethmoid is seen in living leiopelmatids and most microhylids[s7,s9]. *Notobatrachus* was reported to have a paired sphenethmoid[s8], but was later questioned[s5]. We coded this character as "?" in *Notobatrachus*.

28. Vomers: present, paired (0); or absent (1), or present, azygous (2).

    Remarks: Modified from [19] (character 8, 9). The absent condition is a seen in *Xenopus tropicalis*, *Xenopus epitropicalis*, *Hymenochirus* and *Pipa*[19]. The azygous condition is seen in some *Xenopus*[19].

29. Position of anterior process of vomer: anterior process of vomer lying immediately behind premaxilla (0); or lying near premaxilla-maxilla articulation (1).

    Remarks: Modified from [20] (character 23). The condition in *Triadobatrachus* is unknown. In *Notobatrachus*, the anterior plate-like portion of the vomer (anterior process) lies close to the premaxilla, and is more or less parallel to the

cranial midline. In all the other ingroup taxa, the anterior portion of the vomer, if well developed, lies adjacent to the premaxilla-maxilla articulation.

30. Postchoanal process of vomer: absent (0); or present, forming wide angle (about 90-110°) with anterior portion of vomer (1); or present, forming narrow angle (about 45°) with anterior portion of vomer (2).

    Remarks: Modified from [20] (character 24). When present, the postchoanal process of vomer forms the posterior border of the internal choana. Polarity is tentative due to the unknown condition in *Triadobatrachus*. The postchoanal process is absent in *Ascaphus* and independently in *Pelobates*. It is present and forms a wide angle in *Vieraella* and *Notobatrachus*. The angle is significantly narrower in *Leiopelma*, *Bombina*, *Alytes*, *Barbourula,* and *Discoglossus*.

31. Elongation of the postchoanal process of vomer: not elongate (0); or elongate (1).

    Remarks: Modified from [19] (character 11). The derived condition is only seen in *Spea* and *Scaphiopus*.

32. Palatine: present as discrete element (0); or absent (1).

    Remarks: Modified from [19] (character 12) and [20] (character 25). *Triadobatrachus* has palatine retained as a discrete bone. This is considered as the primitive condition. *Neusibatrachus* is coded as absent[s10]. Neobatrachians possess the palatine as a discrete element.

33. Anterior terminus of cultriform process of parasphenoid: extending anteriorly to the level of the vomers (0); or not reaching the level of the vomers (1).

    Remarks: Modified from [20] (character 26). The polarity is tentative due to the unknown condition in *Triadobatrachus*.

34. Posterolateral alae of parasphenoid: anteroposterior width of alae equal or greater than one-third distance between lateral ends (0); or width narrower than one-third distance between lateral ends (1); or alae absent (2).

Remarks: Modified from [20] (character 27). The relatively narrow posterolateral alae are seen in *Triadobatrachus* and considered the primitive condition. The absent condition is seen *Rhinophrynus*, *Xenopus* and *Pipa*.

35. Posterolateral notch of parasphenoid alae: present (0); or absent (1).

Remarks: Modified from [20] (character 28). A notched posterolateral edge is present in *Triadobatrachus*, and is considered the primitive condition. Coding of *Pelodytes caucasicus* as 0 is based on Figure 17 of [S11].

36. Posteromedial process of parasphenoid: absent, leaving the posterior border of parasphenoid straight or concave (0); or present (1).

Remarks: Modified from [20] (character 29). *Triadobatrachus* lacks the posteromedial process of parasphenoid, and this is considered as the primitive condition.

37. Relationships of parasphenoid and sphenethmoid: two elements separate (0); or at least partially fused (1).

Remarks: Modified from [19] (character 33). The derived condition is seen in pipids.

38. Medial ramus of pterygoid: not contacting parasphenoid (0); or contacting parasphenoid (1); or medial ramus absent (2).

Remarks: Modified from [20] (character 30). In *Triadobatrachus*, the pterygoid and parasphenoid do not contact each other[s5], so the absence of contact is

considered as the primitive condition. *Rhinophrynus* is unique in lacking the medial ramus.

39. Ventral flange of the anterior ramus of the pterygoid: absent (0); or present as a ventrally directed flange (1).

    Remarks: Modified from [19] (character 36). The derived condition is seen in *Xenopus*, *Pipa*, *Barbourula*, and *Discoglossus*[19].

40. Parahyoid bone: present and single (0); or present and paired (1); or absent (2).

    Remarks: Modified from [19] (character 170, 171) and [20] (character 31). *Triadobatrachus* has a single parahyoid bone, and this is considered as the primitive condition.

41. Columella: well-ossified columella present (0); or absent (1); or present, but reduced in size (2).

    Remarks: Modified from [19] (character 45, 46) and [20] (character 32). *Triadobatrachus* has an ossified columella preserved[s5] (fig. 3), so the presence of columella is considered as the primitive condition. The derived condition is seen in *Ascaphus*, *Leiopelma*, *Bombina*, *Rhinophrynus* and some neobatrachians. The reduced columella is reported to be present in *Pelobates fuscus* and *P. syriacus*[19].

42. Mentomeckelian bone ossification: present (0); or absent (1).

    Remarks: Modified from [19] (character 66) and [20] (character 33). The absence of mentomackelian is seen in pipoids[19], due to failed ossification of infrarostral cartilage in tadpoles.

43. Upper marginal teeth: present (0); or absent (1).

Remarks: New character. Upper marginal teeth are primitively present in *Triadobatrachus*.

44. Lower marginal teeth: present (0); or absent (1).

    Remarks: New character. Lower marginal teeth are primitively present in *Triadobatrachus.*

45. Occipital foraman: pathway for occipital vessels open on frontoparietal (0); or pathway for occipital vessels roofed by bone (1).

    Remarks: Modified from [13] (character 5). Coding for this character follows [13].

46. Number of presacral vertebrae: 14 or more (0); ten presacral vertebrae (1); normally nine presacral vertebrae (2); normally eight or few (3).

    Remarks: Modified from [20] (character 35).

47. Fusion of presacrals I and II: fusion absent (0); or fusion present (1).

    Remarks: Modified from [19] (character 76) and [20] (character 36). *Pelodytes* is coded as 0[19,s11]. The derived condition is seen in some pipoids and neobatrachians.

48. Centrum of presacral vertebrae: vertebral centra amphicoelous or notochordal (0); or opisthocoelous (1); or procoelous (2).

    Remarks: Modified from [20] (character 37). *Triadobatrachus* has amphicoelous centra, and this is considered as the primitive condition.

49. Neural arch of presacral vertebrae: completely or weakly imbricated roofing of spinal canal (0); or not imbricated with spinal canal partially exposed (1).

    Remarks: Modified from [20] (character 38).

50. Morphology of atlantal cotyles: cotyles mostly ventral and narrowly separated by notochordal fossa (0); cup-like cotyles displaced laterally and widely separated from one another (1); cotyles confluent as a single articular surface (2).

    Remarks: Modified from [20] (character 39).

51. Free ribs on presacral vertebrae: free ribs present on all presacral vertebrae (0); ribs present on presacral II-V or II-VI (1); or ribs restricted to presacrals II-IV (2); or present on presacrals II-IV till subadult stage (3); free ribs absent in both subadults and adults (4).

    Remarks: Modified from [19] (character 80) and [20] (character 40). *Triadobatrachus* has free ribs on all its presacrals and this is considered as the primitive condition.

52. Length of transverse process: transverse process of verbetra II longest, or of equal length of III and IV (0); or transverse process of vertebra III longest (1); or that of IV longest (2).

    Remarks: Modified from [13] (character 40). Condition 2 is seen in living *Pipa* and *Xenopus*.

53. Transverse process of posterior presacral vertebrae: more laterally than anterolaterally oriented (0); or essentially anterolaterally oriented (1).

    Remarks: Modified from [20] (character 41). *Triadobatrachus* has laterally oriented transverse processes on the posterior trunk vertebrae, and this is considered the primitive condition. Most pipoids and pelobatoids (except *Megophrys*) tend to have anterolaterally-oriented transverse processes.

54. Fusion of sacral ribs: remain free from sacral vertebra (0); or fused to transverse process of sacrum (1).

Remarks: Modified from [20] (character 42). *Triadobatrachus* is known to have the sacral ribs free from the sacrum, whereas all the other frogs have the two fused together.

55. Dilation of sacral diapophysis: slender with little or no dilation (0); or moderately dilated and hatchet-shaped, with a convex lateral edge (1); or widely expanded as butterfly wing-shaped, with more or less a straight lateral edge (2).

Remarks: Modified from [19] (character 103) and [20] (character 43). The condition 2 is seen in pipids, *Pelodytes* and *Pelobates*.

56. Postsacral vertebrae: caudal vertebrae remain unfused (0); or urostyle present in association with discrete caudal between sacrum and urostyle (1); or all postsacral vertebrae uniformly modified into single urostyle (2).

Remarks: Modified from [20] (character 44). Six caudal vertebrae are present in *Triadobatrachus*[s5], and the presence of unfused caudal vertebrae is considered as the primitive condition. *Notobatrachus* has one free post-sacral vertebra, and all the other taxa have all the caudal vertebrae fused into the urostyle.

57. Relative length of urostyle: shorter than combined length of presacral vertebrae (0); or as long or longer than combined length of presacral vertebrate (1).

Remarks: Modified from [13] (character 46). *Triadobatrachus* has a short tail, with length significantly shorter than the combined length of the presacral vertebrae. In case of a fused urostyle, it is shorter than the combined length of presacral vertebrae in fossils such as *Vieraella* and *Notobatrachus.* So a short

urostyle is considered as the primitive condition. The polarity is in contrast with [13].

58. Sacral-urostyle articulation: cartilaginous joint (0); bicondylar (1); monocondylar (2); or simply fused (3).

    Remarks: Modified from [19] (character 83) and [20] (character 45). Coding for *Barbarula* and *Bombina* as 2 follows [20]. Coding for *Megophrys* as 0/3 follows [19]. Coding for *Pelobates cultripes* as 2/3 follows specimen CM55769, which has a monocondylar sacral-urostyle articulation.

59. Transverse process on postsacral complex: present (0); or fused to a bony web of sacral diapophysis (1); or absent (2).

    Remarks: Modified from [20] (character 46). Condition 1 is seen in *Spea*.

60. Dorsal Crest on urostyle: absent (0); present, extending to half-length of urostyle (1); present, extending to almost the full length of urostyle (2).

    Remarks: New character based on [S1]. *Notobatrachus* is reported to have a dorsal crest on urostyle[s5], but it is not clear how far it extends back. Therefore we code it as 1/2.

61. Type of pectoral girdle: arciferal, with the epicoracoid cartilages overlapping one another and the sternum attached to the pectoral arch (0); or firmisternal, with the epicoracoid fused to some degree along the midline (1).

    Remarks: Modified from [19] (character 88).

62. Presence of prezonal element: absent (0); or present as a cartilaginous plate (1); or present as a bony style (2).

Remarks: Modified from [19] (character 85). Polarity is tentative due to unknown condition in *Triadobatrachus*. Condition 2 is known in *Megophrys*[19].

63. Posterior ends or epicoracoid cartilages: not expanded (0); or expanded to the level of lateral edge of the sternum (1).

    Remarks: Modified from [19] (character 87). The condition 1 is seen in *Pipa* and *Xenopus*.

64. Length of scapula: at least half-length of humerus (0); or less than half-length of humerus (1).

    Remarks: Modified from [20] (character 47). The primitive condition is known only in *Triadobatrachus*, whereas all the other frogs have a scapula less than half-length of humerus.

65. Overall shape of scapula: short and stocky (0); or relatively long, about two to three times as long as it is wide (1).

    Remarks: Modified from [19] (character 100). *Triadobrachus* has a relatively stocky scapula, and this is considered as the primitive condition. The derived condition is known in *Rhinophrynus*, pelobatoids and neobatrachians. Coding for *Pelodytes punctatus* and *P. ibericus* as 0 follows [S11].

66. Leading edge of scapula: leading edge concave (0); or straight (1).

    Remarks: Modified from [20] (character 48). A concave leading edge of scapula is present in *Triadobatrachus*, and this is considered as the primitive condition.

67. Anterior overlap of clavicle on scapula: overlap absent (0); or overlap present (1); or clavicle and pars acromialis of scapula fused (2).

Remarks: Modified from [20] (character 49). Polarity is tentative due to unknown condition in *Triadobatrachus*. Condition 2 is only known in *Xenopus*.

68. Curvature of long axis of clavicle: straight or only slightly bowed (0); or strongly bowed (1).

    Remarks: New character. *Triadobatrachus* and *Notobatrachus* both have a relatively straight clavicle, and this is considered as the primitive condition.

69. Sternal end of clavicle: narrower than the body of clavicle (0); or sternal end expanded and broader than the body of clavicle (1).

    Remarks: Modified from [19] (character 95). The derived condition is known in *Barbarula* and *Xenopus*[19].

70. Medial end of coracoid: medial end little or slightly expanded, narrower than distal end (0); or medial end of coracoid greatly expanded, wider than the distal end, and usually have an arched edge (1).

    Remarks: Modified from [20] (character 50). Polarity is tentative due to unknown condition in *Triadobatrachus*.

71. Relative lengths of clavicle/coracoid: clavicle approximately equal in length to coracoid (0); or clavicle much longer than coracoid (1).

    Remarks: New character. Polarity is tentative due to unknown condition in *Triadobatrachus*.

72. Cleithrum: present and unforked (0); present and forked (1); or cleithrum fused to suprascapula (2).

Remarks: Modified from [20] (character 51). *Triadobatrachus* has unforked cleithrum, and this is considered as the primitive condition. Coding for *Spea multiplica* as 1 follows [S12] (fig. 8).

73. Bony sternum stylus: absent (0); or present (1).

    Remarks: Modified from [19] (character 91). Polarity is tentative due to unknown condition in *Triadobatrachus*. Condition 1 is known in *Pelobates*, *Megophrys*, *Pelodytes* and some neobatrachians.

74. Condition of sternal plate: sternum absent (0); sternum forming elongate rod (1); sternum forming semicircle with concave anterior margin (2); or sternum forming thin, sickle shape (3).

    Remarks: Modified from [13] (character 49). Polarity is tentative due to unknown condition in *Triadobatrachus*.

75. Humeral condyle: single condyle with small diameter less than 60% of distal width (0); or single condyle enlarged with diameter greater than 60% of distal width (1).

    Remarks: Modified from [20] (character 52).

76. Ossification of humeral condyle: condyle unossified (0); or condyle ossified (1).

    Remarks: Modified from [20] (character 53).

77. Epipodial elements: remaining as separate elements (0); or fused to form single element (1).

    Remarks: Modified from [20] (character 54). The primitive condition is known in *Triadobatrachus* and *Czatkobatrachus*.

78. Free intermedium in carpus: present (0); or absent, by fusion with ulnare (1).

Remarks: Modified from [20] (character 55).

79. Fusion of distal carpal III and IV with postaxial centrale: absent, distal carpals III and IV free (0); or distal carpal IV fused with to postaxial centrale (1); or distal carpal III and IV both fused to postaxial centrale (2).

    Remarks: Modified from [19] (character 135). Condition 1 is known in *Pelodytes*, and condition 2 is known in neobatrachians.

80. Length and orientation of ilium: short ilium essentially dorsally directed (0); or elongate shaft of ilium anteriorly directed (1).

    Remarks: Modified from [20] (character 56). This character is uninformative in our dataset, because all taxa exhibit state 1. It is kept here in case new "intermediate" fossils are found.

81. Dorsal acetabular expansion of ilium: not extending to dorsal limit of ischium (0); or extending to dorsal limit of ischium (1).

    Remarks: Modified from [20] (character 57). According to [20], the derived state is known in *Alytes*, *Discoglossus*, *Rhinophrynus*, and pelobatoids except for *Eopelobates*[s13].

82. Dorsal tubercle of ilium: strongly developed as a distinct tubercle (0); or weakly developed as a low process (1); or essentially absent (2).

    Remarks: Modified from [19] (character 109) and [20] (character 58). *Triadobatrachus* has a prominent dorsal tubercle right before the acetabulum, and this is considered as the primitive condition. In most other anurans, the tubercle is either present as a low prominence or absent.

83. Dorsal crest on body of ilium: absent (0); or present, dorsoventrally directed (1); or present, laterally directed (2).

Remarks: Modified from [19] (character 104). The dorsal crest on the ilium is associated with jumping[s1,s14].

84. Ossification of pubis: pubis remains cartilaginous (0); pubis ossified (1).

Remarks: Modified from [19] (character 111). The derived condition is seen in *Pipa* and *Xenopus* [19].

85. Hind limb proportions: similar or only slightly longer than front limb (0); or proportionally longer (1).

Remarks: Modified from [20] (character 59). The primitive condition is known in *Triadobatrachus*, whereas all the other taxa have the derived condition.

86. Epipubis: absent (0); or present as a large plate (1); or present as a narrow stripe (2).

Remarks: Modified from [19] (character 112). Polarity is tentative due to unknown condition in *Triadobatrachus*. Fossil taxa are all coded as unknown due to the cartilaginous nature of the epipubis.

87. Condition of ventral crest of femur (*crista femoris*): absent or poorly developed (0); or present (1).

Remarks: Modified from [19] (character 115). *Triadobatrachus* lacks a discrete ventral crest on femur, so the absence of ventral crest on femur is considered as the primitive condition. *Ascaphus* and *Leiopelma* are derived in having the ventral crest of femur well developed.

88. Fusion of proximal tarsals: fusion absent (0); or fused at proximal and distal ends (1); or completely fused to form a single bone (2).

Remarks: Modified from [20] (character 60). Tibiale and fibulare is not fused in *Triadobatrachus*, and this is considered as the primitive condition.

89. Number of tarsalia: three or more free elements (0); or only two elements present (1).

Remarks: Modified from [20] (character 61).

90. Prehallux: absent (0); or present as small hind foot element (1); or modified as bony spade (2).

Remarks: Modified from [19] (character 151) and [20] (character 62). Polarity is tentative due to unknown condition in *Triadobatrachus*.

91. Shape of prehallux: sub-oval (0); elongate, scaphoid-shaped (1); or cuneiform-shaped (2).

Remarks: Modified from [19] (character 152).

92. Consolidation of Cranial Nerve V and VII: three separate foramina occur (0); or trigeminal and facial foramina separated by prefacial commissure (1); or commissure absent, nerva exit via single prootic foramen (2).

Remarks: Modified from [20] (character 63).

93. Posture of manus: medial inturning of first finger absent (0); or inturning of the first finger present (1).

Remarks: Modified from [19] (character 133) and [20] (character 65).

94. Depressor mandibulae: consisting of one head or two slightly divided parts witho origin from the dorsal fascia (0); consisting of two discrete bellies that are at least partially separated by the insertion of the cucullaris (1).

    Remarks: Modified from [19] (character 69). Polarity is tentative due to unknown condition in *Triadobatrachus*. Condition 1 is seen in *Xenopus* and *Pipa*[19].

95. Condition of the *depressor mandibulae muscle*: it originates at least in part from the otic region, either from fascia or bone (0); or it originates only from the fascia over the suprascapula (1).

    Remarks: Modified from [19] (character 70). The derived condition is seen in *Pelobates*, *Scaphiopus* and *Pelodytes*[19].

96. Separation of *m. semitendinosus* from *m. sartorius*: *m. sartorius* not completely distinct, at least fused to *m. semitendinosus* to some degree (0); *m. sartorius* completely dinstinct from *m. semitendinosus* (1)

    Remarks: Modified from [19] (character 132).

97. Presence of accessory head of *m. adductor magnus*: abent (0); present (1).

    Remarks: Modified from [19] (character 122).

Supplementary Reference:

1. Reilly, S. M. & Jorgensen, M. E. The evolution of jumping in frogs: morphological evidence for the basal anuran locomotor condition and the radiation of locomotor systems in crown group anurans. *J. Morphol.* **272**, 149-168 (2011).

2. Benson, D. A., Karsch-Mizrachi, I., Lipman, D. J., Ostell, J. & Wheeler, D. L. GenBank. *Nucleic Acids Res.* **33**, D34-D38 (2004).
3. Edgar, R. C. MUSCLE: multiple sequence alignment with high accuracy and high throughput. *Nucleic Acids Res.* **32**, 1792-1797 (2004).
4. Varon, A., Vinh, L. S. & Wheeler, W. C. POY version 4: phylogenetic analysis using dynamic homologies. *Cladistics* **26**, 72-85 (2010).
5. Rocek, Z. & Rage, J. C. in *Amphibian biology, volume 4: palaeontology* (eds H. Heatwole & R. L. Carroll) 1282-1294 (Surrey Beatty & Sons, 2000).
6. Milner, A. R. in *The phylogeny and classification of the tetrapods, volume 1: amphibians, reptiles, birds* (ed M. J. Benton) 59-102 (Clarendon Press, 1988).
7. Trueb, L. in *The Skull, Volume 2: Patterns of Structural and Systematic Diversity* (eds J. Hanken & B. K. Hall) 255-343 (The University of Chicago Press, 1993).
8. Báez, A. & Basso, N. The earliest known frogs of the Jurassic of South America: review and cladistic appraisal of their relationships. *Münch. geowiss. Abh. A Geol. Paläontol.* **30**, 131-158 (1996).
9. Van Eeden, J. A. The development of the chondrocranium of *Ascaphus truei* Stejneger with special reference to the relations of palatoquadrate to the neurocranium. *Acta Zool.* **32**, 41-176 (1951).
10. Baez, A. M. & Sanchiz, B. A review of *Neusibatrachus wilferti*, an Early Cretaceous frog from the Montsec Range, northeastern Spain. *Acta Palaeontol. Pol.* **52**, 477-487 (2007).
11. Sanchiz, B., Tejedo, M. & Sanchez-Herraiz, M. J. Osteological differentiation among Iberian Pelodytes (Anura, Pelodytidae). *Graellsia* **58**, 35-68 (2002).

12. Banbury, B. & Maglia, A. M. Skeletal development of the Mexican spadefoot, *Spea multiplicata* (Anura: Pelobatidae). *J. Morphol.* **267**, 803-821 (2006).

13. Estes, R. New fossil pelobatid frogs and a review of the genus *Eopelobates*. *Bull. Mus. Comp. Zool.* **139**, 293-339 (1970).

14. Emerson, S. B. The ilio-sacral articulation in frogs: form and function. *Biol. J. Linnean Soc.* **11**, 153-168 (1979).
